# Impaired expression of mitochondrial and adipogenic genes in adipose tissue from a patient with acquired partial lipodystrophy (Barraquer-Simons syndrome): a case report

**DOI:** 10.1186/1752-1947-2-284

**Published:** 2008-08-27

**Authors:** Jordi P Guallar, Ricardo Rojas-Garcia, Elena Garcia-Arumi, Joan C Domingo, Eduardo Gallardo, Antoni L Andreu, Pere Domingo, Isabel Illa, Marta Giralt, Francesc Villarroya

**Affiliations:** 1 Departament de Bioquímica i Biologia Molecular and Institut de Biomedicina (IBUB), Universitat de Barcelona, and CIBER Fisiopatologia Obesidad y Nutrición, Instituto de Salud Carlos III, Avda Diagonal 645, 08028, Barcelona, Spain; 2 Servei de Neurologia, Hospital de la Santa Creu i Sant Pau, Antoni Ma Claret 167, 08025, Barcelona, Spain; 3 Departament de Patologia Mitocondrial i Neuromuscular, Institut de Recerca, Hospital Universitari Vall d'Hebron, Pg. Vall d'Hebron 119-129, 08035, Barcelona, Spain; 4 Servei de Medicina Interna, Hospital de la Santa Creu i Sant Pau, Antoni Ma Claret 167, 08025, Barcelona, Spain

## Abstract

**Introduction:**

Acquired partial lipodystrophy or Barraquer-Simons syndrome is a rare form of progressive lipodystrophy. The etiopathogenesis of adipose tissue atrophy in these patients is unknown.

**Case presentation:**

This is a case report of a 44-year-old woman with acquired partial lipodystrophy. To obtain insight into the molecular basis of lipoatrophy in acquired partial lipodystrophy, we examined gene expression in adipose tissue from this patient newly diagnosed with acquired partial lipodystrophy. A biopsy of subcutaneous adipose tissue was obtained from the patient, and DNA and RNA were extracted in order to evaluate mitochondrial DNA abundance and mRNA expression levels.

**Conclusion:**

The expression of marker genes of adipogenesis and adipocyte metabolism, including the master regulator *PPARγ*, was down-regulated in subcutaneous adipose tissue from this patient. Adiponectin mRNA expression was also reduced but leptin mRNA levels were unaltered. Markers of local inflammatory status were unaltered. Expression of genes related to mitochondrial function was reduced despite unaltered levels of mitochondrial DNA. It is concluded that adipogenic and mitochondrial gene expression is impaired in adipose tissue in this patient with acquired partial lipodystrophy.

## Introduction

Acquired partial lipodystrophy (APL) or Barraquer-Simons syndrome is a rare form of progressive lipodystrophy (OMIM 60879). Patients show a progressive and symmetrical lipoatrophy of subcutaneous adipose tissue starting from the face and spreading to the upper part of the body. Other alterations, such as nephropathy or central nervous system abnormalities, are often, but not always, present [1]. Patients without any associated anomalies or medical complications have also been reported [2]. Women are more often affected than men and most of the reported cases appear to be sporadic [3]. However, the appearance of similarly affected relatives in the families of several patients has suggested autosomal dominant inheritance [1,4]. Recently, through a study of 35 cases and extensive review of the literature, Garg and collaborators established a major diagnostic criterion for APL as being gradual onset of bilaterally symmetrical loss of subcutaneous fat from the face, neck, upper extremities and abdomen sparing the lower extremities [5]. It has been reported recently that some cases of APL are associated with mutations in the *lamin B *gene [6], which is reminiscent of other lipodystrophies, such as Dunnigan-type familial partial lipodystrophy type 2, which are associated with *lamin A *mutations [7]. However, the precise mechanisms leading to adipose tissue atrophy in the syndrome remain unknown. Here we present a patient with typical APL and, to obtain insight into the potential etiopathogenic events leading to lipoatrophy in the disease, a gene expression analysis of subcutaneous adipose tissue is reported. The profile of gene expression is compared to the gene expression pattern in subcutaneous fat from healthy control individuals. This study focused on the analysis of the expression of genes characteristic of the adipocyte phenotype, as well as of genes involved in mitochondrial function and local inflammation status in adipose tissue, whose expression is known to be disturbed in experimental models of lipoatrophy as well as in lipodystrophy associated with antiretroviral treatment in patients with HIV.

## Case presentation

The patient was a Caucasian woman from Spain, and who was the first child of non-consanguineous, healthy parents. The neonatal period and her psychomotor development were normal. She had her first menstruation at the age of 13 years, and regularly since then. She has one child following a normal pregnancy history. At age 8 years, she noted that her subcutaneous adipose facial tissue gradually began to decrease and she complained of generalized muscle pain, predominantly in her lower legs, after exercise. The patient was first seen at age 44 years, this being the time at which adipose tissue biopsy was conducted (see below). A physical examination revealed generalized and symmetrical loss of subcutaneous fatty tissue, predominantly in her face and the upper part of her body. The facial lipoatrophy gave an impression of ageing, and a male aspect was noted. However, testosterone levels were normal (0.34 ng/ml, normal range from 0.3 to 1.2 ng/ml). Blood examination showed normal levels of glucose (98 mg/dl, normal range 45 to 135 mg/dl), whereas levels of triglycerides were higher than normal (252 mg/dl, normal range 35 to 150 mg/dl) and levels of cholesterol slightly higher than normal (234 mg/dl, normal range 100 to 220 mg/dl). However, unaltered levels of HDL-cholesterol (47 mg/dl, normal range 35 to 80 mg/dl) and LDL-cholesterol (137 mg/dl, normal range 60 to 150 mg/dl) were found. The blood examination also revealed normal muscular enzyme levels. A neurological examination indicated that deep tendon reflexes were normal and no myotonic phenomena were observed. Nerve conduction studies showed normal values in all tested nerves. A concentric needle examination showed complex repetitive discharges in all tested muscles with no spontaneous activity. Renal and liver function were, as inferred from serum enzyme levels, also normal (ALT/GPT 34 U/liter, normal range 2 to 41 U/liter; AST 27 U/liter, normal range 1 to 40 U/liter; GGT 24 U/liter, normal range 5 to 49 U/liter). Ultrasonic examination of the abdomen indicated hepatic steatosis with normal liver size and morphology, and the kidneys and spleen were normal. Cytogenetic studies revealed a normal karyotype (46XX) without evidence of chromosome breakage. Serum C3 levels were 45 mg/dL, which was abnormally low with respect to normal values (range from 85 to 180 mg/dl). Results were positive for the presence of serum complement 3 nephritic factor. Thus, the overall clinical and biochemical features of the patient led to the diagnosis of APL. The pattern of progressive loss of subcutaneous adipose tissue in the face and the upper part of the body was in accordance with the major criterion established by Misra *et al. *(2004) [5] and supportive criteria were also met: onset during childhood, low serum levels of complement 3 and the presence of serum complement 3 nephritic factor.

A biopsy sample of subcutaneous adipose tissue was taken from the arm. Control values of gene expression in adipose tissue were obtained from the analysis of biopsies of subcutaneous adipose tissue taken from the arms of 10 healthy control individuals (mean age 38.5 ± 9.0 years, 4/6 female/male). The patient and the individual controls gave their consent to participate in the study and the protocol was approved by the Ethics Committee of the Hospital de la Santa Creu i Sant Pau, Barcelona, Spain. Separate analysis of gene expression markers in men and women did not show any significant difference and therefore reference values of gene expression in healthy men and women were shown together. After homogenization in RLT buffer (Qiagen, Hilden. Germany), an aliquot was used for isolation of DNA, which was performed using a standard phenol/chloroform extraction methodology. Another aliquot of the homogenate was used for RNA extraction using a column-affinity based methodology (RNEasy, Qiagen, Hilden, Germany). For mRNA analysis TaqMan Reverse Transcription and -RT-PCR reagents were used (Applied Biosystems, Foster City, CA, USA). One microgram of RNA was transcribed into cDNA using random-hexamer primers and real-time reverse transcriptase-polymerase chain reaction (RT-PCR) was performed on an ABI PRISM 7700HT sequence detection system. The TaqMan RT-PCR was performed in a final volume of 25 μl using TaqMan Universal PCR Master Mix, NoAmpErase UNG reagent and the specific gene expression primer probes. The TaqManGene Expression assays used were: COX4I1 (subunit IV of cytochrome c oxidase, *COIV*), Hs00266371_m1; ATPase5J (subunit F6 of F0-ATPsynthase) Hs0036588_m1; PPARGC1 (*PGC-1α*), Hs0013304_m1; CEBPA (CCAAT/enhancer binding protein-α, *C/EBBP-α*), Hs00269972_s1; PPARG (peroxisome-proliferator activated receptor-γ, *PPAR-γ*), Hs00234592_m1; RB1 (retinoblastoma protein, *pRB*), Hs00153108_m1; DLK1 (*Pref-1*), Hs00171584_m1; UCP2 (uncoupling protein-2, *UCP-2*), Hs00163349_m1; UCP3 (uncoupling protein-3, *UCP-3*), Hs00243297_m1; NRF1 (nuclear respiratory factor-1, *NRF-1*) Hs00192316_m1; LPL (lipoprotein lipase, *LPL*), Hs00173425_m1; LEP (leptin, *LEP*), Hs00174877_m1; Complement precursor-3, Hs00163811_m1; TNF (tumor necrosis factor-α, *TNF-α*), Hs00174128_m1; SLC2A4 (glucose transporter, *GLUT4*), Hs00168966_m1; APM1 (adiponectin), Hs00605917_m1; β2-microglobulin, Hs9999907_m1; MCP-1 (monocyte chemoattractant protein-1, *MCP-1*), Hs00234140_m1; CD68 antigen, Hs00154355. Primers and probe for the detection of cytochrome c oxidase subunit II (*COII*) mRNA and mtDNA abundance were designed as previously reported [8]. Controls with no RNA, primers, or reverse transcriptase were included in each set of experiments. Each sample was run in duplicate and the amount of mRNA for the gene of interest in each sample was normalized to that of the reference control using the comparative (2^-ΔCT^) method. Data for gene transcripts are expressed as the ratio of relative abundance of the mRNA of the gene of interest respect to 18S rRNA (Hs99999901_s1).

Examination of gene expression for master regulatory factors associated with the promotion of adipogenesis indicated that peroxisome proliferator-activated-γ (*PPARγ*) mRNA was the only mRNA significantly down-regulated in the patient with APL with respect to controls (Table 1). The expression of mRNA for another positive regulator of adipogenesis, CCAAT/enhancer binding protein-α (*C/EBPα*) mRNA was not significantly altered. The mRNA levels of retinoblastoma protein pRb, a protein that may have negative effects on adipogenesis [9], and of *Pref-1*, a known negative regulator of adipogenesis [10] were also unchanged. Among adipokines, leptin mRNA levels were unaltered in the patient whereas adiponectin mRNA was down-regulated. The mRNA for two marker genes of adipose tissue metabolism, insulin-sensitive glucose transporter-4 (*GLUT4*) and lipoprotein lipase (*LPL*), were also down-regulated in the patient with respect to controls. In contrast, the mRNA levels for three marker genes of inflammation (tumor necrosis factor-α, *TNFα*; *MCP-1*; and β2-microglobulin) as well as the marker of macrophage infiltration CD68 were unaltered in the patient, with values almost identical to those of controls. Complement component-3 gene expression was also unchanged.

**Table 1:** mRNA expression of genes involved in adipogenesis, metabolism, inflammation and mitochondrial function in adipose tissue from our patient with APL

	Healthy controls	APL	Change
**Adipogenesis (+)**			
*PPARγ *mRNA	2.78 (1.81–3.74) × 10^-5^	1.35 × 10^-5^	↓
*C/EBPα *mRNA	1.55 (0.93–2.03) × 10^-4^	1.66 × 10^-4^	=
**Adipogenesis (-)**			
*pRb *mRNA	7.52 (3.47–11.57) × 10^-6^	3.84 × 10^-6^	=
*Pref-1 *mRNA	0.44 (0.03–0.84) × 10^-9^	0.04 × 10^-9^	=
**Adipokines**			
*Leptin *mRNA	0.73 (0.41–1.02) × 10^-4^	0.85 × 10^-4^	=
*Adiponectin *mRNA	1.05 (0.69–1.39) × 10^-3^	0.49 × 10^-3^	↓
**Metabolism**			
*GLUT4 *mRNA	0.96 (0.43–1.47) × 10^-5^	0.13 × 10^-6^	↓
*LPL *mRNA	2.88 (2.15–3.47) × 10^-4^	0.73 × 10^-4^	↓
**Inflammation**			
*TNFα *mRNA	5.18 (2.74–7.63) × 10^-7^	4.56 × 10^-7^	=
*MCP-1 *mRNA	0.24 (0.07–0.40) × 10^-5^	0.19 × 10^-5^	=
β*2-microglobulin *mRNA	2.85 (2.06–3.49) × 10^-4^	3.12 × 10^-4^	=
*CD68 *mRNA	0.75 (0.26–1.24) × 10^-4^	1.01 × 10^-4^	=
**Complement system**			
*Component 3 mRNA*	1.99 (0.91–3.07) × 10^-4^	2.08 × 10^-4^	=
**OXPHOS**			
*COII *mRNA	1.17 (0.74–1.51) × 10^-2^	0.35 × 10^-2^	↓
*COIV *mRNA	4.24 (3.42–4.81) × 10^-5^	1.55 × 10^-5^	↓
*ATPsynthase F0-6 mRNA*	6.77 (4.85–8.69) × 10^-5^	2.10 × 10^-5^	↓
**UCPs**			
*UCP2 *mRNA	4.05 (2.74–5.77) × 10^-5^	5.72 × 10^-5^	=
*UCP3 *mRNA	4.13 (1.67–6.50) × 10^-7^	2.56 × 10^-7^	=
**Mitochondriogenesis regulators**			
*PGC-1α *mRNA	1.12 (0.71–1.51) × 10^-6^	0.82 × 10^-6^	=
*NRF-1 *mRNA	4.06 (2.96–5.26) × 10^-6^	3.37 × 10^-6^	=

APL, Acquired partial lipodystrophy. Values of mRNA expression in adipose tissue from healthy controls are shown as means (95% confidence interval in parentheses), expressed as the ratio of relative abundance of the mRNA of the gene of interest relative to 18S rRNA. Reduced mRNA expression values in APL below confidence intervals are shown as ↓.

Levels of transcripts corresponding to components of the respiratory chain system (OXPHOS), either mtDNA-encoded (*COII*) or nuclear DNA-encoded (*COIV *and *ATP synthase F0 subunit 6*) were reduced in adipose tissue from the patient with respect to healthy controls. No significant changes were observed for mtDNA abundance in adipose tissue from the patient with respect to reference control values (1.08-fold change with respect to the mean control values). Neither *UCP2 *mRNA levels nor *UCP3 *mRNA levels were altered in the patient relative to controls. Likewise, transcript levels for PPARγ-coactivator-1α *(PGC-1α*) and nuclear respiratory factor-1 (*NRF-1*), regulatory factors of mitochondrial biogenesis, were also unaltered in the patient with respect to controls.

## Discussion

The present study constitutes the first gene expression analysis in adipose tissue from a patient with APL lipodystrophy syndrome and, to our knowledge, the first gene expression analysis of any lipodystrophy disease other than that in highly active antiretroviral-treated (HAART) patients infected with HIV. A clear limitation of the present type of study is that cause-and-effect relationships cannot be established and the observed changes in gene expression could be either a cause or consequence of the alteration in adipose tissue. However, identification of the genes showing altered expression may provide clues for further research into the etiopathogenesis of the disease.

The results indicated that adipose tissue of the patient with APL showed impaired expression of genes associated with the adipocyte differentiation process and this involves genes related to adipose tissue metabolism and *PPARγ*, a master regulatory gene of adipogenesis. The down-regulation of *PPARγ *may be the main causative event of the coordinate impairment in the expression of genes encoding components of adipocyte metabolism or adipokines, as genes such as *GLUT4*, *LPL*, and the adiponectin gene are known targets of *PPARγ*-dependent regulation [11]. PPARγ deficiency due to either haploinsufficiency or to dominant negative activity elicits familial partial lipodystrophy (Dunnigan) type 3 [12] whereas reduced *PPARγ *expression has been observed in fat from patients with HAART-associated lipodystrophy [13,14]. It appears therefore that lowered PPARγ activity, regardless of its origin, may be common to multiple types of lipodystrophy. However, it cannot be excluded that PPARγ down-regulation is an effect, and not a primary cause of the APL syndrome, just as lowered PPARγ gene expression is commonly found in adipose tissue from patients with distinct pathologies leading to lipoatrophy. On the other hand, the absence of a reduction in mRNA for C/EBPα, another master regulator of adipogenesis, does not support an overall impairment of adipogenesis in patients with APL. Unaltered leptin gene expression is consistent with previous observations in patients with APL showing that most of these patients have unaltered circulating levels of leptin [5].

A major difference in the alterations observed in adipose tissue from our patient with respect to subcutaneous fat from lipodystrophic HAART patients concerns marker genes of inflammation. Whereas in HAART patients, the expression of genes related to inflammatory processes are systematically up-regulated [13-15], gene expression for the four markers of inflammation analyzed here is completely normal in our patient. This result does not indicate a major involvement of an inflammatory environment in adipose tissue as causative of lipoatrophy in APL syndrome. However, a wider analysis of markers of inflammation covering the distinct manifestations of the inflammatory process would be required for unequivocal evaluation of the role of inflammation in adipose tissue of patients with APL.

A remarkable finding in this study is the identification of reduced expression of genes for mitochondrial respiratory chain components in adipose tissue from our patient with APL. Mitochondrial impairment has been previously identified in HAART-associated lipodystrophy and it was first attributed to the toxicity of some antiretroviral drugs which cause mitochondrial DNA depletion [16]. However, recent data have established that mitochondrial impairment is a more general phenomenon associated with HAART lipoatrophy, which also involves causative events other than mtDNA depletion [14,17]. The present findings identify for the first time mitochondrial disturbances in adipose tissue from a form of lipoatrophy unrelated to viral infection or antiretroviral treatment, and not associated with mtDNA depletion. This indicates that altered mitochondrial function might be a potential common disturbance in lipoatrophy regardless of its origin, a possibility which deserves further research. Recent findings have indicated a previously unrecognized role of mitochondrial biogenesis in the adipocyte differentiation process [18], and the present observations would fit with mitochondrial impairment as being causative of lipoatrophic phenomena. However, other events related to mitochondrial disturbances, such as mutations in the tRNALys gene of mitochondrial DNA, cause lipomatosis rather than peripheral lipoatrophy [8] and therefore a simple defective mitochondrial activity model cannot solely account for eliciting atrophy in adipose tissue.

## Conclusion

In summary, this is the first gene expression analysis in adipose tissue from a patient with APL. It reveals impaired gene expression for marker genes of adipogenesis, including the master regulator *PPARγ*, and mitochondrial function without signs of altered expression of inflammation-related genes.

## Abbreviations

18S rRNA: 18S ribosomal RNA; APL: Acquired partial lipodystrophy (Barraquer-Simons syndrome); C/EBP-α: CCAAT/enhancer binding protein-α; COIV: subunit IV of cytochrome c oxidase; COII: subunit II of cytochrome c oxidase; *GLUT4*: Glucose transporter-4; HAART: Highly active antiretroviral-treatment; LPL: Lipoprotein lipase; MCP-1: Monocyte chemoattractant protein-1; mtDNA: Mitochondrial DNA; NRF1: Nuclear respiratory factor-1; PGC-1α: peroxisome-proliferator activated receptor-γ co-activator-1α; *PPAR-γ*: Peroxisome-proliferator activated receptor-γ; pRB: retinoblastoma protein; Pref-1: Preadipocyte factor-1; TNF-α: tumor necrosis factor-α; UCP2: Uncoupling protein-2; UCP3: Uncoupling protein-3.

## Competing interests

The authors declare that they have no competing interests.

## Authors' contributions

PD and II analyzed and interpreted the data from the physical examination of the patient and PD was a major contributor in writing the manuscript. RRG, EG and II performed the analysis and interpretation of data in relation to renal and liver function, cytogenetic analysis and blood analysis. EGA and ALA carried out muscle analysis. JCD performed the analysis and interpretation of mitochondrial DNA data and JG and MG analyzed the mRNA. FV designed and coordinated the study and was responsible for final writing of the manuscript. All authors read and approved the final manuscript.

## Consent

Written informed consent was obtained from the patient for publication of this case report. A copy of the written consent is available for review by the Editor-in-Chief of this journal.
